# Evidence of hypervirulent carbapenem-resistant *Klebsiella pneumoniae* in cats with urinary affections and associated humans in Egypt

**DOI:** 10.1038/s41598-025-96147-8

**Published:** 2025-04-15

**Authors:** Sarah M. Hashem, Fatma Abdel-Kader, Elshaimaa Ismael, Ayah M. Hassan, Manar M. Farouk, Mahmoud Elhariri, Rehab Elhelw

**Affiliations:** 1 Department of Microbiology, Immunology and Mycology, Faculty of Veterinary Medicine, Cairo University, Giza, Egypt; 2 Department of Zoonoses, Faculty of Veterinary Medicine, Cairo University, Giza, Egypt; 3 Department of Veterinary Hygiene and Management, Faculty of Veterinary Medicine, Cairo University, Giza, Egypt; 4 Genome Research Unit (GRU), Animal Health Research Institute (AHRI), Dokki, Giza, Egypt; 5 Department of Internal Medicine and Infectious Diseases, Faculty of Veterinary Medicine, Cairo University, Giza, Egypt

**Keywords:** Cats, Carbapenem-resistant, Egypt, Hypervirulent, *Klebsiella pneumoniae*, Phylogenetic tree, Urinary tract infections, Ecology, Microbiology

## Abstract

The emergence of hypervirulent and carbapenem-resistant *Klebsiella pneumoniae* poses a significant threat to the public health of both cats and their owners. Therefore, conducting molecular characterization and phylogenetic analysis of *K. pneumoniae* strains in both cats and humans in Egypt is crucial. 108 feline and 101 human urine samples were collected and subjected to routine microbiological isolation and molecular identification of *K. pneumoniae*. Subsequently, phenotypic antimicrobial sensitivity patterns and molecular identification of classical virulence, hypervirulence, and carbapenem resistance genes were examined. A total of 46 *K. pneumoniae* isolates were recovered, comprising 43.4% (23 out of 53) from diseased humans, 4.17% (2 out of 48) from healthy humans, 22.95% (14 out of 61) from diseased felines, and 14.89% (7 out of 47) from healthy felines. The detection rates for narrow drug-resistant (NDR), multidrug-resistant (MDR), extensively drug-resistant (XDR), and pan drug-resistant (PDR) strains were 41.30%, 54.35%, 2.17%, and 2.17%, respectively. The distribution rates for *mrKD*, *entB*, *K2*, *Kfu*, and *MagA* genes were 76.1%, 82.6%, 8.7%, 13.0%, and 0%, respectively. In addition, the distribution of hypervirulence genes was 41.3%, 36.9%, 13.0%, 10.9%, and 17.4% for *iucA*, *iroB*, *Peg344*, *rmPA*, and *rmPA2*, respectively, and 43.5%, 30.4%, 19.6%, and 52.2% for *NDM*, *OXA-48*, *VIM*, and *KPC* resistance genes, respectively. Phylogenetic analysis of the *entB* gene from four recovered strains revealed a relationship between feline strains and other human strains. In conclusion, this study focused on the molecular characterization and phylogenetic analysis of hypervirulent and carbapenem-resistant *K. pneumoniae* in companion cats and humans in Egypt.

**Supplementary Information:**

The online version contains supplementary material available at 10.1038/s41598-025-96147-8.

## Introduction

The global spread of antimicrobial resistance poses a significant risk to public health, as emphasized by the World Health Organization^1^. *Klebsiella pneumoniae* is regarded as a high-concern pathogen due to its resistance to antimicrobials, necessitating the development of novel control approaches^2,3^. *K. pneumoniae* is a gram-negative, non-motile, encapsulated bacterium that freely colonizes human mucous membranes, including the alimentary tract and oropharynx, with a benign colonization effect. However, *K. pneumoniae* isolates can invade other tissues from these surfaces, leading to severe human infections^4–6^.

The animal-human interface facilitates the cross-species transmission of *K. pneumoniae*, and its zoonotic potential has been documented^7,8^. Interestingly, once the bacterium enters the body, it can exhibit increased virulence and antimicrobial resistance^9,10^. As a result, significant attention has been devoted to *K. pneumoniae* as an infectious pathogen.

The acquisition of new genetic traits leading to the development of antibiotic resistance and hypervirulence raises significant concerns regarding these infections. The emergence of antimicrobial resistance has made the treatment of uncomplicated urinary tract infections more challenging, while *K. pneumoniae*-associated bacteremia and pneumonia have increasingly become life-threatening^11,12^.

Notably, *K. pneumoniae* possesses a distinct set of virulence genes responsible for its pathogenicity, triggering various clinical illnesses^13^. For instance, type 1 fimbriae adhere to human mucosal surfaces, while type 3 fimbriae play a crucial role in biofilm synthesis^14^. Additionally, both the K-capsular antigen and lipopolysaccharides (LPS) are recognized as major virulence factors^15^. Laboratory investigation of hypervirulent *K. pneumoniae* may involve identifying genotypic or phenotypic biomarkers. However, the identification of molecular markers is more precise than phenotypic markers^16^. Hypervirulent *K. pneumoniae* has several genotypic biomarkers that can be used for molecular identification. The most reliable and widely used biomarkers for its detection, with an accuracy of > 5%, include the following five genes: *iucA* (aerobactin siderophore biosynthesis), *iroB* (salmochelin siderophore biosynthesis), *peg-*344 (putative transporter), and *prmpA* and *prmpA2* (regulators of the mucoid phenotype via increased capsule production). The transmission of these molecular biomarkers through plasmids or mobile genetic elements between bacteria is responsible for spreading hypervirulence traits^17^.

Two commonly observed mechanisms contribute to the development of antibiotic resistance in *K. pneumoniae*: extended-spectrum β-lactamases (ESBLs) and carbapenems. ESBLs render bacteria resistant to monobactams and cephalosporins, while carbapenems confer resistance to nearly all available β-lactam antibiotics, including carbapenems^18^. The first expression of carbapenem by *K. pneumoniae* was reported in 1996 in North Carolina, and this type of carbapenem is referred to as *KPC*^19^. Other carbapenems expressed by *K. pneumoniae* strains include *MBL* (metallo-β-lactamases), New Delhi metallo-beta-lactamase (*NDM*-1), Imipenem (*IMP*), and *VIM* (Verona integron-encoded metallo-β-lactamase). Remarkably, *KPC* and these other carbapenems have been expressed by various bacteria and collectively contribute to the global evolution of carbapenem-resistant bacteria^20^.

Meanwhile, the development of multidrug-resistant *K. pneumoniae* requires intensive investigation, as *K. pneumoniae* strains can acquire traits and DNA that enable them to alter their ability to persist in humans and the surrounding environment^20–24^. The expression of antibiotic resistance does not enhance the virulence of *K. pneumoniae* strains, although it makes them more challenging to treat^25,26^.

Furthermore, certain carbapenem-resistant *K. pneumoniae* strains have been found to carry virulence genes, while some hypervirulent strains of *K. pneumoniae* have acquired carbapenem-encoding genes. Both scenarios contribute to the evolution of carbapenem-resistant hypervirulent *K. pneumoniae*^27^. The development of carbapenem-resistant hypervirulent *K. pneumoniae* poses a significant global public health threat due to the severe nature of such strains and the limited treatment options available^28,29^.

Few recent reports have investigated the emergence of hypervirulent carbapenem-resistant *K. pneumoniae* among horses and farm animals in Egypt^8,16^. Cats are the most domesticated pet animals in Egypt, yet literature is scarce focused on bacterial urinary tract diseases in felines caused by *K. pneumoniae* infections in the Egyptian cat population^30^. However, companion animals, such as cats and dogs, have acted as reservoirs for multidrug-resistant (MDR) *K. pneumoniae* associated with severe pathogenicity^31^. This resistance could potentially be transmitted to humans, as the interface between humans and their pets may play a role in the spread and distribution of resistance^32^.

In the Egyptian healthcare system, *K. pneumoniae* is a notorious pathogen. The incidence of MDR and extensively drug-resistant (XDR) isolates has been increasing in intensive care units (ICUs)^33,34^. Therefore, it is essential to fully comprehend the association between antimicrobial resistance factors and phenotypic data and examine the spread of *K. pneumoniae* from a One Health perspective in Egypt^35^. The present study aimed to assess the occurrence of hypervirulent carbapenem-resistant *K. pneumoniae* in cats and humans in contact with them, and to explore the genetic relatedness of the recovered strains between these species.

## Materials and methods

### Ethical approval, study design, and sampled species

All procedures conducted in the current study received approval from the Faculty of Veterinary Medicine, Cairo University-Institutional Animal Care and Use Committee (VETCU-IACUC), under study approval number: Vet CU08072023706. All procedures strictly adhered to the guidelines and regulations and fully complied with the ARRIVE guidelines. Furthermore, for procedures involving human participants, oral informed consent was obtained from all participants after they were informed of the use of urine samples. Ethical approval for using human subjects was obtained from the designated health facility (National Research Centre, Giza, Egypt), and all procedures were performed following the Declaration of Helsinki.

A cross-sectional pilot study was conducted, targeting a convenient sample size of cats and humans in Giza Governorate, Egypt. Informed consent was obtained from the targeted individuals and pet owners before sampling. The inclusion criteria for cats included any age or sex, and they could be either healthy or afflicted with urinary tract conditions such as complete or incomplete urine retention, with or without bloody urine, stranguria, or renal failure, and admitted to veterinary clinics. The inclusion criteria for the human population encompassed individuals admitted to hospitals, those attending private laboratories for urine analysis, and pet owners in contact with cats. All included individuals either suffered from urinary tract conditions or were healthy. Cats and humans residing outside of Giza Governorate were excluded from the study.

### Sampling and transportation

The sampling process was conducted from August 2022 to December 2022. A total of 209 urine samples were collected, comprising 101 from individuals and 108 from cats. Among the sampled individuals were 20 pet owners, 10 hospitalized patients, 68 individuals attending private laboratories, and 3 veterinarians, with 53 classified as diseased and 48 as apparently healthy. The 108 targeted cats were sourced from veterinary clinics.

Urine samples were collected from humans using the clean catch midstream method, which involved cleaning the external genitalia, followed by voiding and collecting midstream urine after several milliliters had passed without flow interruption. Cat collection methods varied based on their health status: midstream urine was obtained during catheterization for cats with complete urine retention, while midstream samples were collected by gentle massage of the urinary bladder and urethra for cats with incomplete urine retention and healthy ones.

All urine samples were collected in sterile cups and clearly labeled with the identification number of the cats or individuals, the date, and the time of collection. The samples were promptly transported in ice boxes to the Laboratory of Microbiology at the Faculty of Veterinary Medicine, Cairo University, for further processing.

### Isolation and identification of *Klebsiella* spp

After receiving the urine samples, all cups were gently mixed, and approximately 1 mL from each urine sample was inoculated into brain heart infusion (BHI) broth (Oxoid, UK) and incubated at 37 °C for 24 h to promote *Klebsiella* propagation. Meanwhile, the inoculated samples were plated on eosin methylene blue (EMB) agar medium (Oxoid, UK) and incubated for 24 h at 37 °C. The presumptive 2–3 mm circular, convex, pink to purple-colored, mucoid, and translucent to opaque colonies were purified by subculturing on EMB agar plates and biochemically identified based on the urea hydrolysis test, citrate utilization test, and carbohydrate fermentation test using urea agar base, Simmon’s citrate agar, and triple sugar iron (TSI) agar media (Oxoid, UK), respectively^36,37^. After routine biochemical identification, presumptive *Klebsiella* spp. isolates were propagated in BHI broth and incubated overnight at 37 °C. The cultured broths were subsequently analyzed.

### Molecular identification and characterization of virulence and carbapenem-resistant encoding genes of the recovered *Klebsiella* spp. isolates

#### DNA extraction from the recovered *Klebsiella* spp. isolates

DNA extraction from the preserved isolates was conducted using the boiling method^38,39^. Briefly, 1.5 mL of single colony-inoculated BHI broth was incubated overnight at 37 °C and then pelleted for 10 min at 1200 ×g. The pellets were resuspended in 150 µL of sterile distilled water and lysed for 15 min at 100 °C in a heat thermo-blocker. Subsequently, pelleting was performed by centrifugation for 10 min at 1200 ×g, and the bacterial DNA in the supernatant was used as a DNA template for PCR.

#### Molecular identification of *Klebsiella spp* isolates

The *gyrA* gene is one of the highly conserved genes selected for the molecular identification of *Klebsiella* genus isolates using uniplex polymerase chain reaction (PCR). Meanwhile, multiplex PCR was used to identify the species of the confirmed *Klebsiella* genus isolates, utilizing the *magA* and *pehX* genes for *K. pneumoniae* and *K. oxytoca* species, respectively^40^.

#### Determination of classical virulence genes and molecular identification of hypervirulent strains

Five of the most critical virulence genes, including mannose-resistant *Klebsiella*-like hemagglutinin D (*mrKD*), enterobactin B (*entB*), capsular serotype gene K2 (*K2*), *Klebsiella* ferric uptake (*Kfu*), and microviscosity-associated gene A (*MagA*), were detected using multiplex PCR. Furthermore, to identify the hypervirulent strains, five uniplex PCR reactions were performed on *K. pneumoniae* isolates to detect the presence or absence of hypervirulence biomarkers, including aerobactin siderophore biosynthesis (*iucA*), salmochelin siderophore biosynthesis (*iroB*), putative transporter (*peg-344*), regulator of mucoid phenotype A (*rmPA*), and regulator of mucoid phenotype A2 (*rmPA2*). The isolates were considered hypervirulent if they harbored at least one of these hypervirulence biomarker genes^16^. Additionally, all molecularly identified hypervirulent isolates were phenotypically confirmed by conducting the string test, as described by Fang et al.^41^. The formation of a viscous string > 5 mm long was considered a positive result.

#### Determination of carbapenem-resistant encoding genes

One multiplex and two uniplex PCR reactions were performed to investigate the distribution of class D oxacillinase-48 (*OXA-48*), carbapenemase (*KPC*), New Delhi metallo-beta-lactamase (*NDM*), and Verona integron metallo-beta-lactamase (*VIM*) resistance genes in *Klebsiella* strains. A multiplex PCR was conducted to detect *KPC* and *NDM* genes, while two uniplex PCR reactions were applied to determine the presence of each of the *VIM* and *OXA-48* genes.

#### PCR conditions

PCR was performed in a total 25 µL reaction mixture, comprising 5 µL of DNA template, 5 µL of 5× TaqMaster mix (Jena Bioscience, Germany), 1 µL of each forward and reverse primer (10 pmol/µL), with the volume adjusted to 25 µL using PCR-grade water (Jena Bioscience, Germany). After electrophoresis using 1.5% (wt/vol) agarose stained with 0.5 µg/mL ethidium bromide, the amplified PCR products were visualized under a UV illumination system. Gel images were captured with a GelDoc 1000 fluorescent imaging system (Bio-Rad) and analyzed with Gel-Pro Analyzer^®^ version 4 (Media Cybernetics, Silver Spring, MD, USA). The PCR cycling conditions, primer sets, and amplicon size for each gene are consistent with previously conducted studies^40,42–46^.

### Phenotypic antibiotic sensitivity test and determination of multiple antimicrobial resistance (MAR) index

The susceptibility of *Klebsiella* isolates to the following antibiotics was examined using the Kirby-Bauer disc diffusion method on Mueller–Hinton agar: cefoxitin (FOX, 10 µg), cefpodoxime (CPD, 10 µg), cefotaxime (CTX, 30 µg), ceftazidime (CAZ, 30 µg), ceftriaxone (CRO, 30 µg), cefepime (CPM, 10 µg), aztreonam (ATM, 30 µg), meropenem (MEM, 10 µg) and ertapenem (ETP, 10 µg) of β-lactams class, amikacin (AK, 30 µg) and gentamycin (CN, 30 µg) of aminoglycoside class, ciprofloxacin (CIP, 5 µg) and nalidixic acid (NA, 30 µg) of quinolones class, Trimethoprim/sulphamesoxazole 1:19 (SXT, 25 µg) of Sulphonamide/ complex class, chloramphenicol (C, 30 µg) of phenicol class, tetracycline (TE, 30 µg) of tetracycline class, and azithromycin (AZM, 30 µg) of macrolides class. The results were interpreted according to the breakpoint recommendations of the Clinical and Laboratory Standards Institute (CLSI 2020)^47^. The MAR index of each *Klebsiella* isolate was assessed using the formula of Paul et al.^48^, where the number of antibiotics to which isolates expressed resistance was divided by the total number (9) of antibiotics to which the isolate had been evaluated for susceptibility. The MAR index results were used to classify isolates according to Christopher et al.^49^ as follows: isolates with an index < 0.3 are considered to have narrow drug resistance (NDR); those with an index ≥ 0.3–0.7 are classified as MDR; an index of 0.8–0.9 indicates XDR isolates; and pan drug-resistant (PDR) isolates expressed resistance to all tested antimicrobials.

### Sequencing and phylogenic analysis of *EntB* gene

Four identified *K. pneumoniae* strains were selected for phylogenetic analysis, comprising three hypervirulent carbapenem-resistant MDR strains and one hypervirulent NDR strain. The targeted gene for sequence analysis was the *entB* gene, the most predominant virulence gene in the recovered strains. The chosen strains were recovered from both human and feline species, and each strain’s profile is presented in Supplementary Table 1. Phylogenetic analysis was performed to establish the relationship between the investigated strains and test the hypothesis of their transmission from one host to another. The amplicons (400 bp) of the *entB* gene were purified using a QIAquick purification kit (QIAGEN, Germany) and partially sequenced using a BigDye Terminator V3.1 sequencing kit (Applied Biosystems). The sequenced fragments were blasted in GenBank and compared with available sequences in the public domain using the NCBI BLAST server. Publicly accessible sequences on NCBI GenBank were retrieved, downloaded, and aligned with CLUSTALW in BioEdit software version 7.0.1.4.

Evolutionary phylogenetic analysis was inferred by using the Maximum Likelihood method and Tamura 3-parameter model^50^. The tree with the highest log likelihood (− 444.37) is shown. The percentage of trees in which the associated taxa clustered together is shown next to the branches. Initial tree(s) for the heuristic search were obtained automatically by applying Neighbor-Join and BioNJ algorithms to a matrix of pairwise distances estimated using the Tamura 3 parameter model, and then selecting the topology with superior log likelihood value. The tree is drawn to scale, with branch lengths measured in the number of substitutions per site. This analysis involved 17 nucleotide sequences. Codon positions included were 1st + 2nd + 3rd + Noncoding. There were a total of 273 positions in the final dataset. Tamura 3 parameter was selected as the best nucleotide substitiution model that would fit our dataset as suggested by the “find best DNA model” tool implemented in MEGA. Phylogenetic analysis was conducted using MEGA11 software^51^ with an estimated bootstrap values < 50 was shown next to nodes.

### Statistical analysis

Data were summarized and presented as percentages (%). Chi-square (χ^2^), Fisher’s exact, and tetrachoric correlation tests were used to calculate the correlation between *Klebsiella* prevalence and various variables. Statistical analysis was performed using PASW Software, Version 18.0 (SPSS Inc., Chicago, IL, USA). Plots were generated in R (Version 3.6.1, R Foundation for Statistical Computing) using the ‘ggplot2’ and ‘ggpubr’^52,53^ packages, as well as the ‘pheatmap’^54^ package. Tetrachoric correlations were performed using the ‘psych’^55^ library. Significance was set at the 0.05 level (P-value).

## Results

### Occurrence of *K. pneumoniae* in cats and contact humans

Forty-six *Klebsiella* sp. isolates were recovered and identified via biochemical reactions and molecular PCR assay targeting the *Klebsiella* genus-specific *gyrA* gene. All 46 recovered isolates were classified as *K. pneumoniae* via multiplex PCR assay, with all samples being positive for the *magA* gene and negative for the *pehX* gene. Of the 46 isolated *K. pneumoniae*, 25 (24.8%) isolates were from humans and 21 (20%) from cats. The prevalence of *K. pneumoniae* was 4.2%, 43.4%, 14.9%, and 22.9% in apparently healthy humans, diseased humans, apparently healthy cats, and diseased cats, respectively (Table 1).

**Table 1:** Prevalence of *K. pneumoniae*.

Species	Human			Feline		
Status	Clinically diseased (admitted to hospitals and laboratories)	Apparently healthy (cat’s owner)	Total	Clinically diseased	Apparently healthy	Total
Total number	53	48	101	61	47	108
Positive samples	23	2	25	14	7	21
Prevalence	43.4%	4.2%	24.8%	22.9%	14.9%	20%

### Distribution of carbapenem-resistant and virulence genes of *K. pneumoniae*

Different uniplex and multiplex PCR assays were performed to detect the carbapenem-resistant genes and virulence genes harbored by *K. pneumoniae*. 28 diverse virulence gene profiles, 12 resistance gene profiles, and 34 hypervirulence carbapenem-resistant gene profile combinations were detected among the recovered *K. pneumoniae* isolates (Supplementary Tables 2, 3, and 4). Moreover, the recovered *K. pneumoniae* isolates were classified based on their carbapenem resistance and harbored virulence genes as follows: 63.0% were carbapenem-resistant hypervirulent *K. pneumoniae* (CrHvKP), 17.4% were non-carbapenem-resistant hypervirulent *K. pneumoniae* (NCrHvKP), 13.0% were carbapenem-resistant non-hypervirulent classical virulent *K. pneumoniae* (CrNHvCvKP), and 2.2% for each of carbapenem-resistant non-hypervirulent non-classical virulent *K. pneumoniae* (CrNHvNCvKP), non-carbapenem-resistant non-hypervirulent classical virulent *K. pneumoniae* (NCrNHvCvKP), and non-carbapenem-resistant non-hypervirulent non-classical virulent *K. pneumoniae* (NCrNHvNCvKP) according to Mohammed et al.^56^.

Based on the characterization of virulence and resistance genes, the distribution of the investigated virulence genes within *K. pneumoniae* isolates was 82.6%, 76.1%, 41.3%, 36.9%, 17.4%, 13.0%, 13.0%, 10.9%, 8.7%, and 0% for *entB*, *mrKD*, *iucA*, *iroB*, *rmPA2*, *peg344*, *Kfu*, *rmPA*, *K2*, and *MagA*, respectively. Meanwhile, the distribution of investigated resistance genes was 52.2%, 43.5%, 30.4%, and 19.6% for *KPC*, *NDM*, *OXA-48*, and *VIM*, respectively (Supplementary Table 4).

### The antimicrobial susceptibility and multiple antimicrobial resistance (MAR) index of the isolated strains

**Fig. 1:**
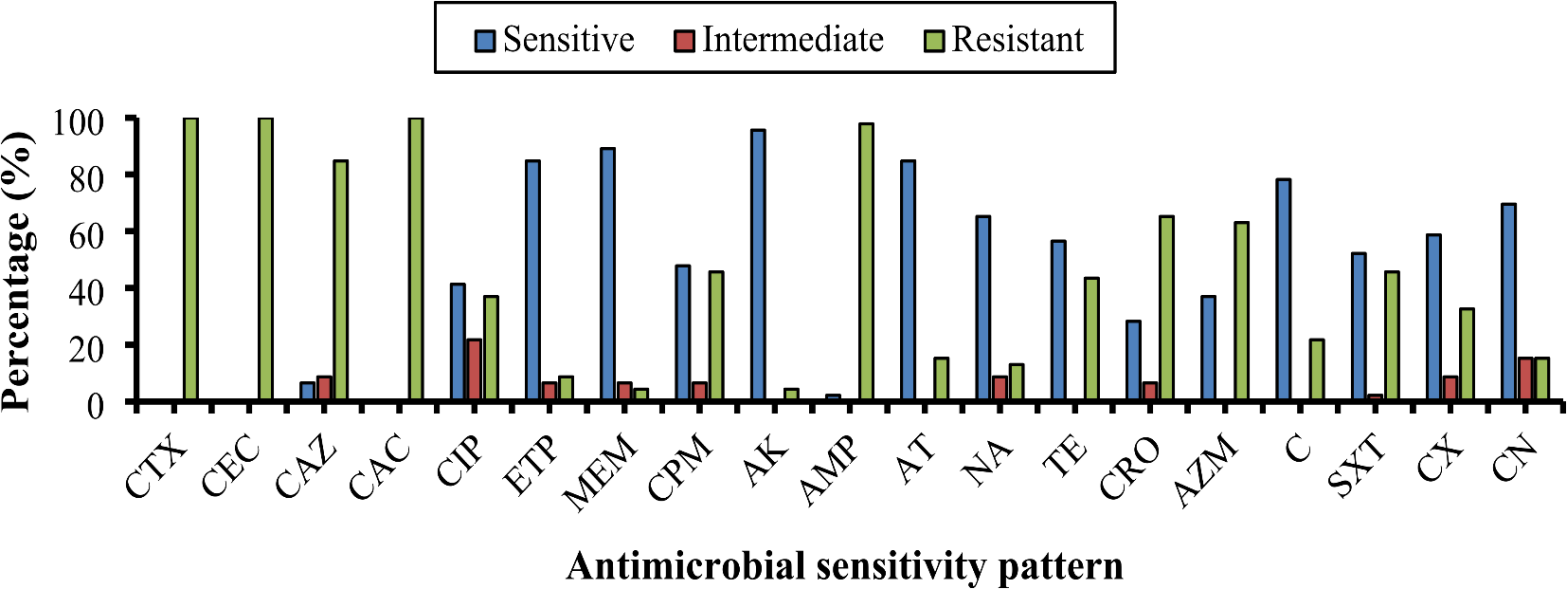
Overall antimicrobial sensitivity patterns of the recovered *K. pneumoniae* isolates from both humans and felines.

**Fig. 2:**
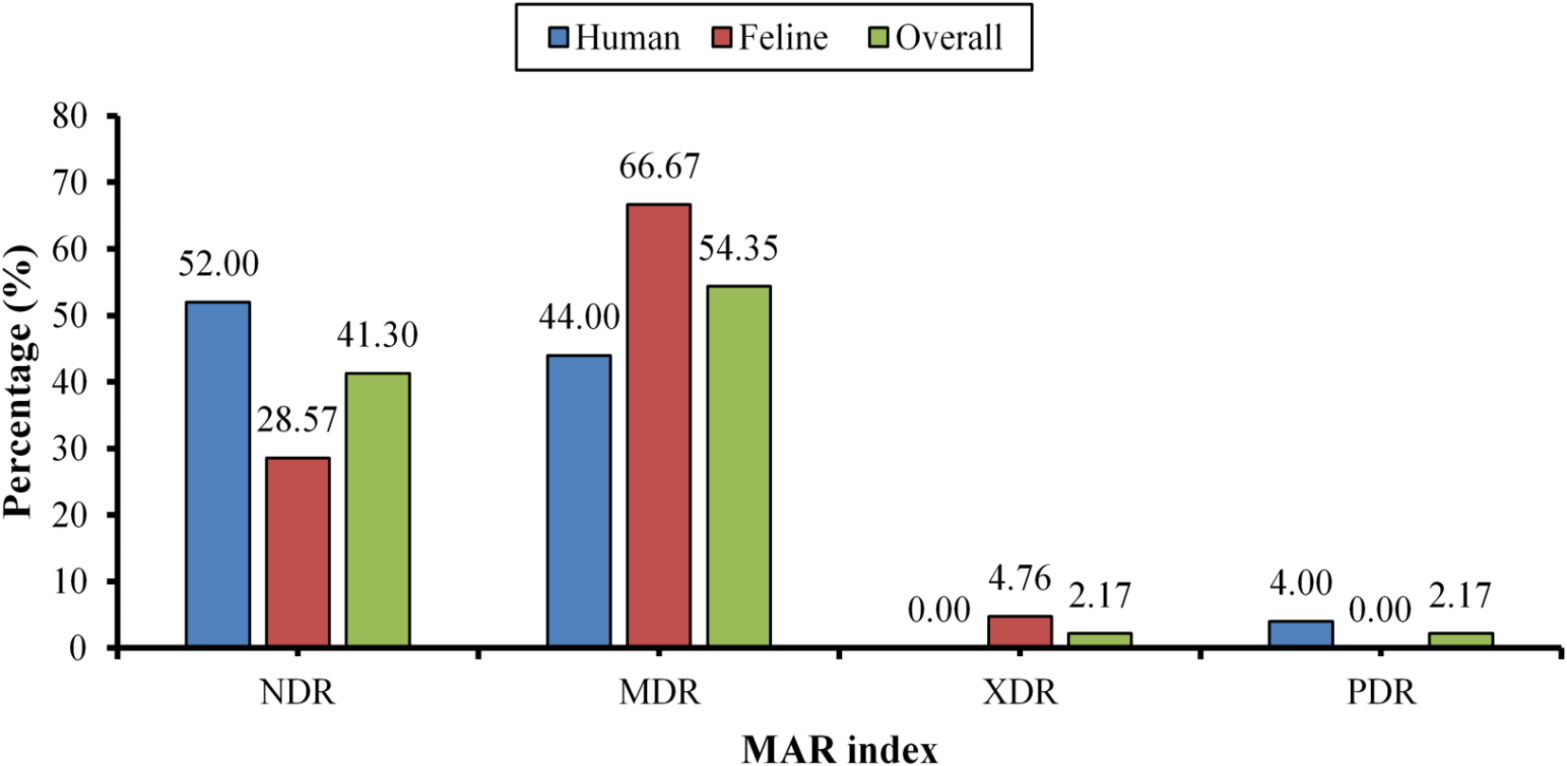
Classification of the recovered *K. pneumoniae* isolates according to the MAR index.

The phenotypic antimicrobial susceptibility testing of the recovered *K. pneumoniae* isolates revealed that the highest resistance was exhibited towards ampicillin (AMP, 97.8%). The decreasing order of resistance percentages against the tested antibiotics was as follows: ceftazidime (CAZ, 84.8%), ceftriaxone (CRO, 65.2%), azithromycin (AZM, 63.0%), cefepime (CPM, 45.7%), Trimethoprim/sulphamesoxazole 1:19 (SXT, 45.7%), tetracycline (TE, 43.5%), ciprofloxacin (CIP, 36.9%), cefotaxime (CTX, 32.6%), cefpodoxime (CPD, 26.1%), chloramphenicol (C, 21.7%), aztreonam (ATM, 15.2%), gentamycin (CN, 15.2%), nalidixic acid (NA, 13.0%), ertapenem (ETP, 8.7%), amikacin (AK, 4.4%) and meropenem (MEM, 4.4%) (Supplementary Tables 6 and Fig. 1). Furthermore, the classification of the recovered *K. pneumoniae* isolates according to the MAR index revealed that 54.4%, 41.3%, 2.2%, and 2.2% were identified as MDR, NDR, XDR, and PDR, respectively (Supplementary Tables 7, 8 and Fig. 2).

### The tetrachoric correlation and heat map associations analysis

The tetrachoric correlation analysis revealed a coefficient of 0.11 between the host type and *Klebsiella* isolation rate, indicating a weak association (*P* > 0.05). However, correlations of − 0.49 (for all hosts) and − 0.75 (for humans) were observed between host health status and *Klebsiella* isolation rate, suggesting a relatively strong association (*P* < 0.0001). No association was observed between host type and MAR index of *Klebsiella* isolates.

**Fig. 3:**
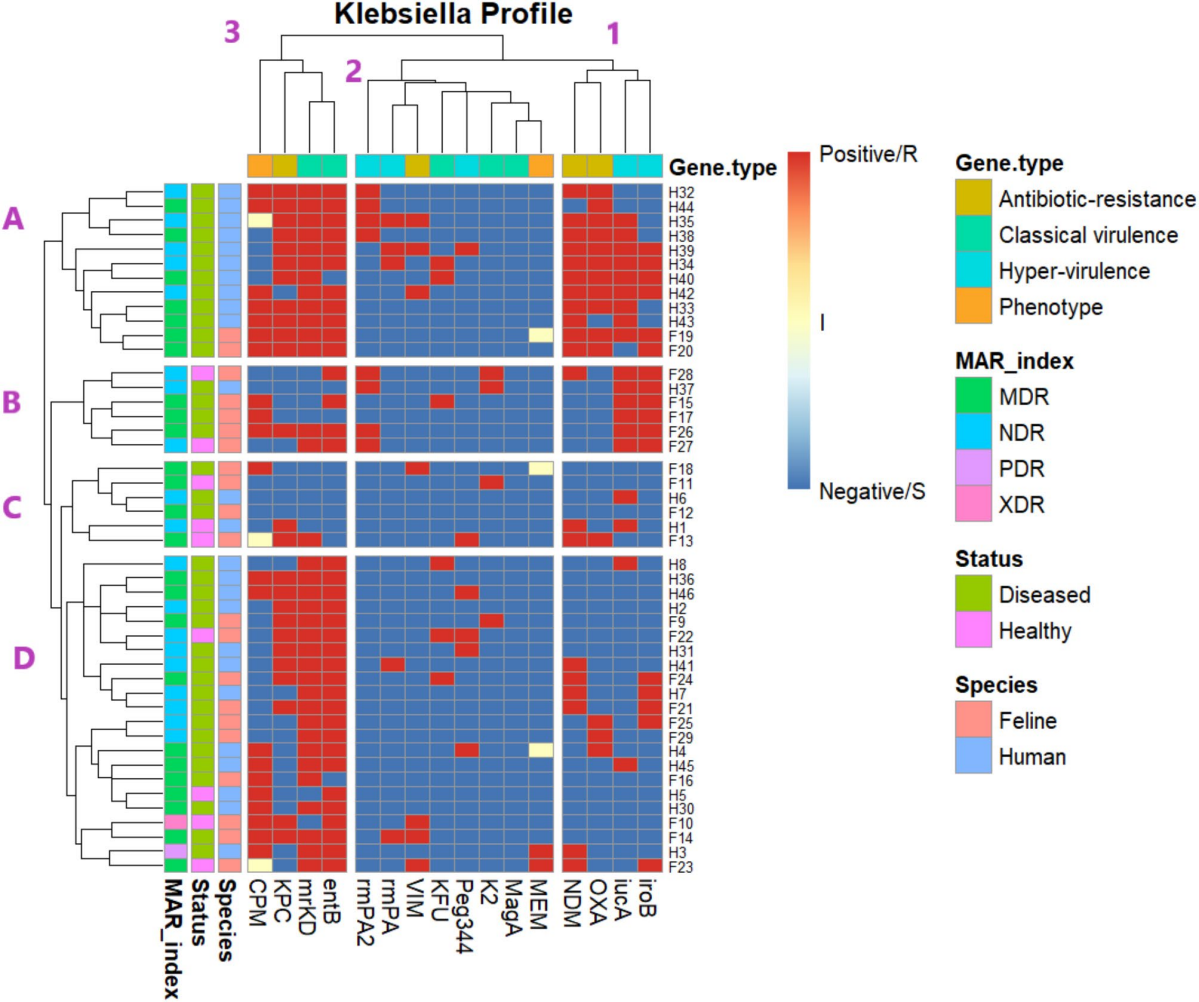
Hierarchically clustered heatmap showing the distribution of antibiotic resistance genes and virulence genes of 46 *Klebsiella* strains isolated from feline (*n* = 21) and human (*n* = 25) hosts, with varied health statuses (healthy and diseased). The map plotted the hyper-virulence genes (iucA, iroB, Peg344, rmPA, rmPA2), classical-virulence genes (mrKD, entB, K2, KFU, MagA), and antibiotic-resistance genes (NDM, OXA, VIM, KPC) as positive (red) and negative (blue). The phenotypic classifications (MEM and CPM sensitivities) were plotted as sensitive (S) as blue, intermediate (I) as yellow, and resistant (R) as red.

Figure 3 shows the hierarchical heat map, which grouped host samples into four clusters (A, B, C, and D). Cluster A mainly gathered *Klebsiella* isolates from diseased humans, which exhibited MAR indices of MDR and NDR and positivity for hypervirulence genes (*iucA* and *iroB*), classical virulence genes (*mrKD* and *entB*), antibiotic-resistance genes (*NDM*, *OXA*, and *KPC*), and cefepime (CPM) phenotype. This observation is compatible with the strong tetrachoric correlation between humans’ health status and *Klebsiella* isolation rate (− 0.75; *P* < 0.001). Cluster D combined *Klebsiella* isolates from diseased and healthy humans and felines and displayed positivity for classical virulence genes (*mrKD* and *entB*), antibiotic-resistance genes (*KPC*), and cefepime (CPM) phenotype. This is compatible with the weak tetrachoric correlation between the health status of felines and the *Klebsiella* isolation rate (− 0.18; *P* = 0.294).

### Phylogenetic analysis of a partial codon sequence of the *EntB* gene

**Fig. 4:**
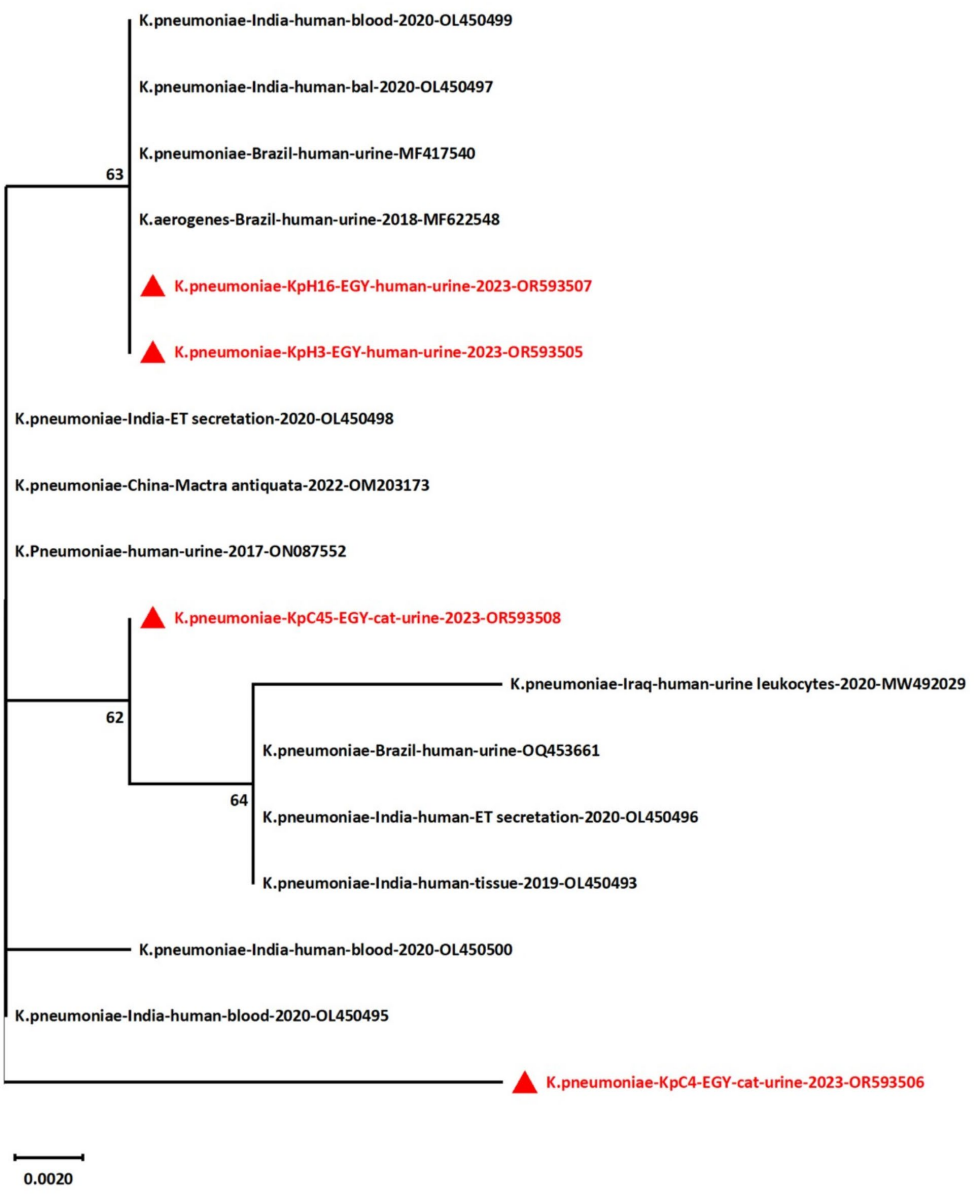
Maximum Likelihood tree showing the relationship between the nucleotide sequences of the partial codon sequences of the *entB* gene. The bootstrap values < 50 was shown next to nodes. The study strains are red in color labeled with red triangles.

The GenBank deposition of the partial codon sequences of four *entB* genes was conducted. The accession numbers of the deposited sequences are OR593505, OR593506, OR593507, and OR593508. Sequencing and phylogeny analyses of the *entB* gene revealed the similarity of the study strains to other *K. pneumoniae* strains obtained from GenBank and the relatedness of cat isolates to human strains, as displayed in Fig. 4. Sequence analysis revealed that the four strains were distributed across three clusters. OR593507 and OR593505 (human strains, urine source) were in the same cluster, exhibiting polytomies with each other and other sequences recovered from Indian human blood (OL450499 and OL450497) and Brazilian human urine (MF622548 and MF417540), respectively. OR593508 (cat strain) clustered closely with other retrieved sequences isolated from human blood, urine, tissue and endotracheal (ET) secretion in various locations, including India (OL450493, tissue and OL450496, endotracheal (ET) secretion), Brazil (OQ453661, urine), and Iraq (MW492029, urine). Additionally, OR593506 (cat strain) formed a single cluster with close relations to other analyzed sequences.

## Discussion

Despite significant progress in companion animal medicine, *K. pneumoniae* infections continue to pose a risk to the health of cats and their owners. *K. pneumoniae* is a major infectious etiology of urinary tract problems in humans and animals. In the present study, 46 *Klebsiella* strains were identified as *K. pneumoniae*.

At minimum, it is essential to differentiate between *Klebsiella* species such as *K. variicola*, *K. quasipneumoniae*, and *K. pneumoniae*, as these species have been identified in both wild and companion animals, whereas *K. oxytoca* is found in humans. In this study, all recovered human and feline isolates tested positive for the conserved *magA* gene of *K. pneumoniae* and negative for the *pehX* gene of *K. oxytoca*. Therefore, further PCR reactions to exclude *K. variicola* and *K. quasipneumoniae* in feline isolates were deemed unnecessary.

The recovered strains were categorized into six groups: carbapenem-resistant hypervirulent *K. pneumoniae* (CrHvKP); carbapenem-resistant non-hypervirulent classical virulent *K. pneumoniae* (CrNHvCvKp); carbapenem-resistant non-hypervirulent non-classical virulent *K. pneumoniae* (CrNHvNCvKp); non-carbapenem-resistant hypervirulent *K. pneumoniae* (NCrHvKp); non-carbapenem-resistant non-hypervirulent classical virulent *K. pneumoniae* (NCrNHvCvKp); and non-carbapenem-resistant non-hypervirulent non-classical virulent *K. pneumoniae* (NCrNHvNCvKp) according to Mohammed et al.^56^. The virulence and carbapenem resistance gene combinations were arranged into 34 patterns (Supplementary Table 2).

The expression of both hypervirulence and carbapenem resistance genes in the same strains may be due to the co-localization of these genes on the same plasmid^57^. The presence of such strains in diseased cases complicates clinical management and increases the risk of lethal nosocomial infections^58^. Although antimicrobial-resistant hypervirulent *K. pneumoniae* strains are infrequently detected worldwide^59–61^, they appear to be more predominant in cats and humans in Egypt.

The results of the present study revealed a weak association between host type and isolation rate but a strong association between host health status and the *K. pneumoniae* isolation rate. Furthermore, no association was observed between host type and the MAR index of these isolates. Accordingly, the emergence of MDR bacteria is expected to increase morbidity and mortality rates, prolong hospitalization, and escalate treatment costs^62^.

The recovered *K. pneumoniae* isolates exhibited varying degrees of resistance to the tested antimicrobials, with resistance rates of 97.8% to AMP and 4.4% to MEM and AK. This resistance may be attributed to the development of extended-spectrum beta-lactamase (ESBL) resistance mechanisms^63^. The distribution of the investigated resistance genes was 52.2% for *KPC*, 43.5% for *NDM*, 30.4% for *OXA-48*, and 19.6% for *VIM*. Multiple resistance genes were detected in the recovered isolates, with some harboring different resistance genes simultaneously. This finding aligns with Ali and Omer^64^ and Satir^65^, who reported various resistance genes in their strains.

The most prevalent virulence gene among the *K. pneumoniae* isolates was *entB*, which is responsible for the siderophore system of *K. pneumoniae* and was present in 82.6% of all recovered isolates (92% in humans and 71.4% in felines) closely resembling the findings of Albasha et al.^66^.

Additionally, *mrkD* is a crucial virulence gene that plays a vital role in adhesion, as reported by Chen et al.^67^. The *mrkD* gene was detected in 76.1% of all recovered isolates, with 84% in human isolates and 66.7% in feline isolates. These results are consistent with Albasha et al.^66^, who reported this gene in 78.3% of all recovered *K. pneumoniae* isolates. The variation in these results may be attributed to the mode of acquisition of this plasmid-mediated gene, as described by Aljanaby and Alhasani^68^, demonstrating the restricted spread of the *rmpA* gene in locally recovered *K. pneumoniae* strains in Egypt.

The *rmpA* gene synthesizes capsular polysaccharides, establishes a mucoid phenotype, and enhances *K. pneumoniae* resistance to bactericidal action, all characteristic of virulent *K. pneumoniae*. The virulence-promoting gene *peg-344* on the virulence plasmid is widely distributed among hypervirulent *K. pneumoniae* strains. The expression product of *peg-344* is predicted to function as an inner membrane transporter. Although *peg-344* is essential for optimal virulence in the in vivo models, it has minimal impact on systemic infection. Similarly, the *iucA* gene, which is responsible for iron acquisition and aerobactin siderophore assembly, contributes to the enhanced virulence and pathogenicity of *K. pneumoniae*^69,70^.

Moreover, the *kfu* gene was detected in 13% of all isolates. This finding contrasts with the study by Albasha et al.^65^, in which the gene was present in 60% of isolates. Additionally, the *magA* gene was absent in all recovered isolates in the present study, whereas it was detected in 13.3% of isolates in the study by Albasha et al.^66^.

The capsular *K2* gene was found in 8.7% of all recovered isolates. This result differs from the findings of Albasha et al.^66^, in which the gene was present in 51.7% of isolates. This discrepancy may be attributed to the presence of other capsular serotypes in the isolates, as suggested by Ho^71^.

Finally, the isolates were classified into 28 and 12 combination profiles based on the distribution of virulence and resistance genes, respectively. This finding highlights the diversity of resistance and virulence genes in the investigated isolates. The variation in the distribution of these genes may be attributed to differences in the geographical origins of the studied isolates. Furthermore, the multiplicity of virulence genes in HpVkp strains underscores the severe pathogenic nature of these isolates and their public health significance. Hypervirulent isolates can cause a pyogenic liver abscess (PLA), bloodstream infections, hospital-acquired pneumonia, intra-abdominal infections, and other illnesses in humans^58^.

Statistical analyses of the study findings revealed no significant difference between apparently healthy and diseased individuals regarding resistance and virulence genes. Specifically, there was no significant difference in the number of resistance genes between healthy and diseased cats (*P* > 0.05). In contrast, a significant difference was observed in the number of resistance genes between healthy and diseased humans (*P* < 0.0001). Additionally, human samples exhibited distinct clustering, with apparently healthy individuals predominantly grouped in cluster B, along with a strong tetrachoric correlation between human health status and the *Klebsiella* isolation rate (− 0.75; *P* < 0.001). Moreover, Pearson correlation coefficients indicated a weak correlation between the virulence and resistance genes of *Klebsiella* isolated from feline and human hosts.

The study analyzed partial codon sequences of the *entB* gene from four *K. pneumoniae* strains and 22 isolates retrieved from GenBank to conduct a robust phylogenetic analysis. The *entB* gene was selected due to its widespread distribution in severe human cases of endocarditis and pneumonia caused by *K. pneumoniae* infections. The aim was to investigate the phylogenetic relationship between the recovered human and feline strains and human strains retrieved from GenBank based on the *entB* gene sequence.

The constructed tree elucidated the relatedness of the recovered feline and human strains to other virulent human strains from different locations, underscoring the potential pathogenicity and virulence of the study strains in humans and the possible role of companion cats’ urine as a source of emerging *K. pneumoniae* infections in their owners. The evolution of CrHvKP among felines presents a significant public health concern. It highlights the potential role of felines in the epidemiology of these resistant and hypervirulent strains through fecal shedding, which may contaminate food, water, and the surrounding human environment, as well as the possible direct zoonotic transmission to the human gut, leading to unrestricted person-to-person transmission^72^.

As a result, stringent preventive and control measures, such as daily cleaning of litter boxes, are imperative to prevent contamination of the food chain in both humans and animals. The dissemination of bacterial pathogens like *K. pneumoniae* between cats and humans poses a risk of transmission to individuals in contact, such as pet owners and veterinarians. Notably, the phylogenetic analysis in this study identified potential preventive and control measures for *K. pneumoniae* infections in both cats and humans.

tudy highlighted the potential emergence of new, highly pathogenic, hypervirulent, carbapenem-resistant *K. pneumoniae* strains in both cats and humans and underscored the possible spread of these strains across different hosts and localities. Accordingly, accurate control and preventive measures should be implemented to mitigate their transmission among companion cats, pet owners, and other exposed individuals.

## Electronic supplementary material

Below is the link to the electronic supplementary material.

Supplementary Material 1

## Data Availability

All data generated or analyzed in this study are included in this published article. The raw sequence data reported in this paper have been deposited in the National Center for Biotechnology Information (NCBI) under the accession numbers OR593505, OR593506, OR593507, and OR593508, and are publicly accessible at the following links: https://www.ncbi.nlm.nih.gov/nuccore/OR593505, https://www.ncbi.nlm.nih.gov/nuccore/OR593506, https://www.ncbi.nlm.nih.gov/nuccore/OR593507, and https://www.ncbi.nlm.nih.gov/nuccore/OR593508.

## Abbreviations

BHI: Brain heart infusion
EMB: Eosin methylene blue
entB: Enterobactin B
ESBLs: Extended-spectrum B-lactamases
HV: Hypervirulent
IMP: Imipenem
K2: Capsular serotype gene K2
Kfu: Klebsiella ferric uptake
KPC: Carbapenem
MagA: Mucoviscosity-associated gene A
MDR: Multidrug resistance
mrKD: Mannose-resistant Klebsiella like hemoagglutinin D
NDM: New Delhi metallo-betalactamase
NDR: Narrow drug resistance
OXA-48: Class D oxacillinase-48
PCR: Polymerase chain reaction
PDR: Pan drug resistance
PLA: Pyogenic liver abscess
rmPA: Regulatory mucoid phenotype A
TSI: Triple sugar iron
VETCU-IACUC: Veterinary Medicine, Cairo University-Institutional Animal Care and Use Committee
