# Supplementary material for: Evidence of hypervirulent carbapenem-resistant Klebsiella pneumoniae in cats with urinary affections and associated humans in Egypt
Source: Sci Rep. 2025 Apr 15;15:12950. doi: 10.1038/s41598-025-96147-8 (PMC12000467; doi:10.1038/s41598-025-96147-8)
Supplement: Supplementary file 1 — Supplementary Material 1 [file 41598_2025_96147_MOESM1_ESM.docx]

**Supplementary Table 1. Details about the selected *K. pneumoniae* strains used for sequencing and phylogenic analysis**

| **Sample ID Number** | 30 | 74 | 58 | 101 |
| --- | --- | --- | --- | --- |
| **Host** | Feline Diseased | Human Diseased | Human Diseased | Feline Diseased |
| **MAR index** | MDR | NDR | MDR | MDR |
| **Virulo-resistance Profile** | *mrKD-entB-iucA-iroB-NDM-OXA-VIM* | *mrKD-entB-iucA-rmPA-rmPA2-NDM-OXA-VIM-KPC.* | *mrKD-entB-Kfu- iucA-iroB- NDM-OXA-KPC.* | *mrKD-entB-K2- iucA KPC.* |
| **Virulence*Resistance resistance profile number** | 11 | 25 | 17 | 33 |
| **Virulence profile number** | 4 | 12 | 16 | 21 |
| **Resistance profile number** | 7 | 10 | 7 | 3 |

**Supplementary Table 2. Distribution of virulence and resistance genes profiles combinations of *K.*** *pneumoniae* **strains isolates for identification of HvCr *K. pnumonia***

| **Profile** | **Resistance Genes** | | | | **Hyper Virulence Genes** | | | | | **Total**  **(46)** | **Human (25)** | | **Feline (21)** | |
| --- | --- | --- | --- | --- | --- | --- | --- | --- | --- | --- | --- | --- | --- | --- |
|  | ***NDM*** | ***OXA*** | ***VIM*** | ***KPC*** | ***iucA*** | ***iroB*** | ***Peg-344*** | ***rmpA*** | ***rmpA2*** |  | **Apparent Healthy**  **(2)** | **Diseased**  **(23)** | **Apparent Healthy**  **(7)** | **Diseased**  **(14)** |
| **1** | + | - | - | - | - | - | - | - | - | 1 | 0 | 1 | 0 | 0 |
| **2** | + | - | - | - | - | + | - |  | - | 1 | 0 | 1 | 0 | 0 |
| **3** | + | - | - | - | + | + | - | - | + | 1 | 0 | 0 | 1 | 0 |
| **4** | - | + | - | - | - | - | + | - | - | 1 | 0 | 1 | 0 | 0 |
| **5** | - | + | - | - | - | + | - | - | - | 1 | 0 | 0 | 0 | 1 |
| **6** | - | + | - | - | - | - | - | - | - | 1 | 0 | 0 | 0 | 1 |
| **7** | - | - | - | + | - | - | - | - | - | 1 | 0 | 1 | 0 | 0 |
| **8** | - | - | - | + | - | - | + | - | - | 3 | 0 | 2 | 1 | 0 |
| **9** | - | - | + | - | - | - | - | - | - | 1 | 0 | 0 | 0 | 1 |
| **10** | + | - | - | + | + | - | - | - | - | 2 | 1 | 1 | 0 | 0 |
| **11** | + | - | - | + | - | + | - | - | - | 2 | 0 | 0 | 0 | 2 |
| **12** | + | - | - | + | - | - | - | + | - | 1 | 0 | 1 | 0 | 0 |
| **13** | - | - | + | + | - | - | - | - | - | 1 | 0 | 1 | 0 | 0 |
| **14** | - | - | + | + | - | - | - | + | - | 1 | 0 | 0 | 0 | 1 |
| **15** | - | - | + | + | + | + | - | - | - | 1 | 0 | 0 | 0 | 1 |
| **16** | + | + | - | + | - | - | + | - | - | 1 | 0 | 0 | 1 | 0 |
| **17** | + | + | - | + | + | + | - | - | - | 2 | 0 | 1 | 0 | 1 |
| **18** | + | + | - | + | - | + | - | - | - | 1 | 0 | 0 | 0 | 1 |
| **19** | + | + | - | + | - | - | - | - | + | 1 | 0 | 1 | 0 | 0 |
| **20** | + | + | - | + | + | - | - | - | - | 1 | 0 | 1 | 0 | 0 |
| **21** | + | + | - | + | + | + | - | + | - | 1 | 0 | 1 | 0 | 0 |
| **22** | + | + | - | + | + | - | - | - | + | 1 | 0 | 1 | 0 | 0 |
| **23** | + | - | + | - | - | + | - | - | - | 1 | 0 | 0 | 1 | 0 |
| **24** | - | + | - | + | - | - | - | - | + | 1 | 0 | 1 | 0 | 0 |
| **25** | + | + | + | + | + | - | - | + | + | 1 | 0 | 1 | 0 | 0 |
| **26** | + | + | + | + | + | + | + | + | - | 1 | 0 | 1 | 0 | 0 |
| **27** | + | + | + | - | + | + | - | - | - | 1 | 0 | 1 | 0 | 0 |
| **28** | - | - | - | - | - | - | - | - | - | 4 | 1 | 1 | 1 | 1 |
| **29** | - | - | - | - | + | - | - | - | - | 3 | 0 | 3 | 0 | 0 |
| **30** | - | - | - | - | + | + | - | - | - | 2 | 0 | 0 | 0 | 2 |
| **31** | - | - | - | - | + | + | - | - | + | 2 | 0 | 1 | 1 | 0 |
| **32** | - | - | + | + | + | - | - | - | - | 1 | 0 | 0 | 1 | 0 |
| **33** | - | - | - | + | + | - | - | - | - | 1 | 0 | 0 | 0 | 1 |
| **34** | - | - | - | - | - | - | + | - | - | 1 | 0 | 0 | 0 | 1 |

**Supplementary Table 3. Distribution of virulence genes profile combinations of *K.*** *pneumoniae* **strains isolates from diseased and apparently healthy feline and human**

| **Profile** | **Virulence Genes** | | | | | | | | | | **Total**  **(46)** | **Human** | | **Feline** | |
| --- | --- | --- | --- | --- | --- | --- | --- | --- | --- | --- | --- | --- | --- | --- | --- |
|  | ***mrKD*** | ***entB*** | ***K2*** | ***Kfu*** | ***MagA*** | ***iucA*** | ***iroB*** | ***Peg-344*** | ***rmpA*** | ***rmpA2*** |  | **Apparent Healthy** | **Diseased** | **Apparent Healthy** | **Diseased** |
| **1** | - | + | - | - | - | - | - | - | - | - | 1 | 1 | 0 | 0 | 0 |
| **2** | + | + | - | - | - | - | - | - | - | - | 5 | 0 | 4 | 0 | 1 |
| **3** | + | + | - | - | - | + | - | - | - | - | 3 | 0 | 3 | 0 | 0 |
| **4** | + | + | - | - | - | + | + | - | - | - | 2 | 0 | 1 | 0 | 1 |
| **5** | + | + | - | - | - | - | - | + | - | - | 3 | 0 | 2 | 1 | 0 |
| **6** | + | + | - | - | - | - | - | - | - | + | 2 | 0 | 1 | 0 | 1 |
| **7** | + | + | - | - | - | - | + | - | - | - | 5 | 0 | 1 | 1 | 3 |
| **8** | + | + | - | - | - | - | - | - | + | - | 2 | 0 | 1 | 0 | 1 |
| **9** | + | + | - | - | - | + | - | - | - | + | 1 | 0 | 1 | 0 | 0 |
| **10** | + | + | - | - | - | + | + | - | - | + | 2 | 0 | 1 | 1 | 0 |
| **11** | + | + | - | - | - | + | + | + | + | - | 1 | 0 | 1 | 0 | 0 |
| **12** | + | + | - | - | - | + | - | - | + | + | 1 | 0 | 1 | 0 | 0 |
| **13** | - | - | - | - | - | + | - | - | - | - | 1 | 0 | 0 | 0 | 1 |
| **14** | - | - | - | - | - | + | + | - | - | - | 1 | 0 | 0 | 0 | 1 |
| **15** | - | - | - | - | - | - | - | - | - | - | 2 | 0 | 1 | 0 | 1 |
| **16** | + | + | - | + | - | + | - | - | - | - | 1 | 0 | 1 | 0 | 0 |
| **17** | + | + | - | + | - | - | + | - | - | - | 1 | 0 | 0 | 0 | 1 |
| **18** | + | + | - | + | - | + | + | - | - | - | 1 | 0 | 1 | 0 | 0 |
| **19** | + | + | - | + | - | + | + | - | + | - | 1 | 0 | 1 | 0 | 0 |
| **20** | + | + | - | + | - | - | - | + | - | - | 1 | 0 | 1 | 0 | 0 |
| **21** | + | + | + | - | - | + | - | - | - | - | 1 | 0 | 0 | 0 | 1 |
| **22** | + | - | - | - | - | - | - | + | - | - | 1 | 0 | 0 | 1 | 0 |
| **23** | + | - | - | - | - | - | - | - | - | - | 1 | 0 | 0 | 0 | 1 |
| **24** | - | - | + | - | - | - | - | - | - | - | 2 | 0 | 1 | 1 | 0 |
| **25** | - | + | + | - | - | + | + | - | + | - | 1 | 0 | 0 | 1 | 0 |
| **26** | - | + | - | - | - | + | - | - | - | - | 1 | 0 | 0 | 1 | 0 |
| **27** | - | + | - | - | - | - | - | - | - | - | 1 | 1 | 0 | 0 | 0 |
| **28** | - | + | - | + | - | - | - | - | - | - | 1 | 0 | 0 | 0 | 1 |

**Supplementary Table 4. Distribution of resistance genes profile combinations of *K.*** *pneumoniae* **strains isolates from diseased and apparently healthy feline and human**

| **Profile** | **Resistance Genes** | | | | **Total**  **(46)** | **Human** | | **Feline** | |
| --- | --- | --- | --- | --- | --- | --- | --- | --- | --- |
|  | ***NDM*** | ***OXA-48*** | ***VIM*** | ***KPC*** |  | **Apparent Healthy** | **Diseased** | **Apparent**  **Healthy** | **Diseased** |
| **1** | + | - | - | - | 2 | 0 | 1 | 1 | 0 |
| **2** | - | + | - | - | 3 | 0 | 1 | 0 | 2 |
| **3** | - | - | - | + | 5 | 0 | 3 | 1 | 1 |
| **4** | - | - | + | - | 1 | 0 | 0 | 0 | 1 |
| **5** | + | - | - | + | 7 | 1 | 3 | 0 | 3 |
| **6** | - | - | + | + | 4 | 0 | 1 | 1 | 2 |
| **7** | + | + | - | + | 7 | 0 | 4 | 1 | 2 |
| **8** | + | - | + | - | 1 | 0 | 0 | 1 | 0 |
| **9** | - | + | - | + | 1 | 0 | 1 | 0 | 0 |
| **10** | + | + | + | + | 2 | 0 | 2 | 0 | 0 |
| **11** | + | + | + | - | 1 | 0 | 1 | 0 | 0 |
| **12** | - | - | - | - | 12 | 1 | 5 | 2 | 4 |

**Supplementary Table 5. Distribution of different virulence and resistance genes in the investigated *K. pneumoniae* strains**

| **Genes** | **Number (Percentage) of the investigated *K.*** *pneumoniae* **strains** | | | | | | **Total**  **(46)** |
| --- | --- | --- | --- | --- | --- | --- | --- |
|  | **Human (25)** | | | **Feline (21)** | | |  |
|  | **Apparent Healthy 2** | **Diseased 23** | **Total 25** | **Apparent Healthy 7** | **Diseased 14** | **Total 21** |  |
| **Virulence Genes Distribution** | | | | | | | |
| ***mrKD*** | 0 (0) | 21 (91.3) | 21 (84) | 4 (57.1) | 10 (71.4) | 14 (66.7) | 35 (76.1) |
| ***entB*** | 2 (100) | 21 (91.3) | 23 (92) | 5 (71.4) | 10 (71.4) | 15 (71.4) | 38 (82.6) |
| ***K2*** | 0 (0) | 1 (4.3) | 1 (4) | 2 (28.6) | 1 (7.1) | 3 (14.3) | 4 (8.7) |
| ***Kfu*** | 0 (0) | 3 (13.0) | 3 (12) | 1 (14.3) | 2 (14.3) | 3 (14.3) | 6 (13.0) |
| ***MagA*** | 0 (0) | 0 (0) | 0 (0) | 0 (0) | 0 (0) | 0 (0) | 0 (0) |
| ***iucA*** | 1(50) | 12 (52.2) | 13 (52) | 2 (28.6) | 4 (28.6) | 6 (28.6) | 19 (41.3) |
| ***iroB*** | 0 (0) | 6 (26.1) | 6 (24) | 3 (42.9) | 8 (57.1) | 11 (52.4) | 17 (36.9) |
| ***Peg344*** | 0 (0) | 4 (17.4) | 4 (16) | 2 (28.6) | 0 (0) | 2 (9.5) | 6 (13.0) |
| ***rmPA*** | 0 (0) | 4 (17.4) | 4 (16) | 0 (0) | 1 (7.1) | 1 (4.8) | 5 (10.9) |
| ***rmPA2*** | 0 (0) | 5 (21.7) | 5 (20) | 2 (28.6) | 1 (7.1) | 3 (14.3) | 8 (17.4) |
| **Resistance Genes Distribution** | | | | | | | |
| ***NDM*** | 1 (50) | 11 (47.8) | 12 (48) | 3 (42.9) | 5 (35.7) | 8 (38.1) | 20 (43.5) |
| ***OXA*** | 0 (0) | 9 (39.1) | 9 (36) | 1 (14.3) | 4 (28.6) | 5 (23.8) | 14 (30.4) |
| ***VIM*** | 0 (0) | 4 (17.4) | 4 (16) | 2 (28.6) | 3 (21.4) | 5 (23.8) | 9 (19.6) |
| ***KPC*** | 0 (0) | 13 (56.5) | 13 (52) | 3 (42.9) | 8 (57.1) | 11 (52.4) | 24 (52.2) |

**Supplementary Table 6. Antimicrobial phenotypic sensitivity pattern of the recovered *K. pnumoniae* isolates**

| **Antimicrobial** | **Human isolates**  **25 (%)** | | | **Feline isolates**  **21 (%)** | | | **Total**  **46 (%)** | | |
| --- | --- | --- | --- | --- | --- | --- | --- | --- | --- |
|  | **S** | **I** | **R** | **S** | **I** | **R** | **S** | **I** | **R** |
| **CAZ** | **3**  **(12%)** | **1**  **(4%)** | **21**  **(84%)** | **0**  **(0%)** | **3**  **(14.29%)** | **18**  **(85.71%)** | **3**  **(6.52%)** | **4**  **(8.70%)** | **39**  **(84.78%)** |
| **CIP** | **12**  **(48%)** | **6**  **(24%)** | **7**  **(28%)** | **7**  **(33.33%)** | **4**  **(19.05%)** | **10**  **(47.62%)** | **19**  **(41.30%)** | **10**  **(21.74%)** | **17**  **(36.96%)** |
| **ETP** | **21**  **(84%)** | **1**  **(4%)** | **3**  **(12%)** | **18**  **(85.71%)** | **2**  **(9.52%)** | **1**  **(4.76%)** | **39**  **(84.78%)** | **3**  **(6.52%)** | **4**  **(8.70%)** |
| **MEM** | **23**  **(92%)** | **1**  **(4%)** | **1**  **(4%)** | **18**  **(85.71%)** | **2**  **(9.52%)** | **1**  **(4.76%)** | **41**  **(89.13%)** | **3**  **(6.52%)** | **2**  **(4.35%)** |
| **CPM** | **12**  **(48%)** | **1**  **(4%)** | **12**  **(48%)** | **10**  **(47.62%)** | **2**  **(9.52%)** | **9**  **(42.86%)** | **22**  **(47.83%)** | **3**  **(6.52%)** | **21**  **(45.65%)** |
| **AK** | **23**  **(92%)** | **0**  **(0%)** | **2**  **(8%)** | **21**  **(100%)** | **0**  **(0%)** | **0**  **(0%)** | **44**  **(95.65%)** | **0**  **(0%)** | **2**  **(4.35%)** |
| **AMP** | **1**  **(4%)** | **0**  **(0%)** | **24**  **(96%)** | **0**  **(0%)** | **0**  **(0%)** | **21**  **(100%)** | **1**  **(2.17%)** | **0**  **(0%)** | **45**  **(97.83%)** |
| **AT** | **21**  **(84%)** | **0**  **(0%)** | **4**  **(16%)** | **18**  **(85.71%)** | **0**  **(0%)** | **3**  **(14.29%)** | **39**  **(84.78%)** | **0**  **(0%)** | **7**  **(15.22%)** |
| **NA** | **19**  **(76%)** | **2**  **(8%)** | **4**  **(16%)** | **11**  **(52.38%)** | **2**  **(9.52%)** | **8**  **(38.10%)** | **30**  **(65.22%)** | **4**  **(8.70%)** | **6**  **(13.04%)** |
| **TE** | **18**  **(72%)** | **0**  **(0%)** | **7**  **(28%)** | **8**  **(38.1%)** | **0**  **(0%)** | **13**  **(61.90%)** | **26**  **(56.52%)** | **0**  **(0%)** | **20**  **(43.48%)** |
| **CRO** | **8**  **(32%)** | **2**  **(8%)** | **15**  **(60%)** | **5**  **(23.81%)** | **1**  **(4.76%)** | **15**  **(71.43%)** | **13**  **(28.26%)** | **3**  **(6.52%)** | **30**  **(65.22%)** |
| **CPD** | **19**  **(76%)** | **2**  **(8%)** | **4**  **(16%)** | **11**  **(52.38%)** | **2**  **(9.52%)** | **8**  **(38.10%)** | **30**  **(65.22%)** | **4**  **(8.70%)** | **12**  **(26.09%)** |
| **AZM** | **10**  **(40%)** | **0**  **(0%)** | **15**  **(60%)** | **7**  **(33.33%)** | **0**  **(0%)** | **14**  **(66.67%)** | **17**  **(36.96%)** | **0**  **(0%)** | **29**  **(63.04%)** |
| **C** | **22**  **(88%)** | **0**  **(0%)** | **3**  **(12%)** | **14**  **(66.67%)** | **0**  **(0%)** | **7**  **(33.33%)** | **36**  **(78.26%)** | **0**  **(0%)** | **10**  **(21.74%)** |
| **SXT** | **13**  **(52%)** | **0**  **(0%)** | **12**  **(48%)** | **11**  **(52.38%)** | **1**  **(4.76%)** | **9**  **(42.86%)** | **24**  **(52.17%)** | **1**  **(2.17%)** | **21**  **(45.65%)** |
| **CX** | **17**  **(68%)** | **3**  **(12%)** | **5**  **(20%)** | **10**  **(47.62%)** | **1**  **(4.76%)** | **10**  **(47.62%)** | **27**  **(58.70%)** | **4**  **(8.70%)** | **15**  **(32.61%)** |
| **CN** | **23**  **(92%)** | **1**  **(4%)** | **1**  **(4%)** | **9**  **(42.86%)** | **6**  **(28.57%)** | **6**  **(28.57%)** | **32**  **(69.57%)** | **7**  **(15.22%)** | **7**  **(15.22%)** |

**Supplementary Table 7. Phenotypic antimicrobial resistance pattern of the recovered isolates**

| **Species/ Status** | **Isolate ID** | **Antibiogram results**  **(17 antimicrobial agents)** | | | **Number of the tested antimicrobials to which the isolates are resistant** | **MAR**  **index value** | **MAR index pattern** |
| --- | --- | --- | --- | --- | --- | --- | --- |
|  |  | **S** | **I** | **R** |  |  |  |
| **Apparently healthy human** | **75** | **11** | **0** | **6** | **6** | **0.35** | **MDR** |
|  | **86** | **14** | **1** | **2** | **2** | **0.12** | **NDR** |
| **Clinically diseased human** | **3** | **16** | **0** | **1** | **1** | **0.05** | **NDR** |
|  | **6** | **15** | **0** | **2** | **2** | **0.12** | **NDR** |
|  | **12** | **13** | **0** | **4** | **4** | **0.24** | **NDR** |
|  | **19** | **10** | **2** | **5** | **5** | **0.29** | **NDR** |
|  | **21** | **10** | **2** | **5** | **5** | **0.29** | **NDR** |
|  | **24** | **9** | **1** | **7** | **7** | **0.41** | **MDR** |
|  | **27** | **15** | **0** | **2** | **2** | **0.12** | **NDR** |
|  | **30** | **14** | **0** | **3** | **3** | **0.18** | **NDR** |
|  | **37** | **5** | **0** | **12** | **12** | **0.71** | **MDR** |
|  | **47** | **11** | **0** | **6** | **6** | **0.35** | **MDR** |
|  | **55** | **9** | **1** | **7** | **7** | **0.41** | **MDR** |
|  | **56** | **7** | **2** | **8** | **8** | **0.47** | **MDR** |
|  | **57** | **9** | **1** | **7** | **7** | **0.41** | **MDR** |
|  | **58** | **7** | **3** | **7** | **7** | **0.41** | **MDR** |
|  | **61** | **9** | **0** | **8** | **8** | **0.47** | **MDR** |
|  | **65** | **7** | **0** | **10** | **10** | **0.59** | **MDR** |
|  | **66** | **15** | **0** | **2** | **2** | **0.12** | **NDR** |
|  | **73** | **15** | **1** | **1** | **1** | **0.05** | **NDR** |
|  | **74** | **10** | **2** | **5** | **5** | **0.29** | **NDR** |
|  | **91** | **0** | **0** | **17** | **17** | **1.00** | **PDR** |
|  | **98** | **11** | **2** | **4** | **4** | **0.24** | **NDR** |
|  | **99** | **10** | **1** | **6** | **6** | **0.35** | **MDR** |
|  | **101** | **12** | **1** | **4** | **4** | **0.24** | **NDR** |
| **Apparently healthy feline** | **8** | **10** | **3** | **4** | **4** | **0.24** | **NDR** |
|  | **20** | **6** | **3** | **8** | **8** | **0.47** | **MDR** |
|  | **28** | **13** | **2** | **2** | **2** | **0.12** | **NDR** |
|  | **47** | **13** | **2** | **2** | **2** | **0.12** | **NDR** |
|  | **82** | **8** | **0** | **14** | **14** | **0.82** | **XDR** |
|  | **95** | **8** | **0** | **9** | **9** | **0.53** | **MDR** |
|  | **108** | **10** | **1** | **6** | **6** | **0.35** | **MDR** |
| **Clinically diseased feline** | **17** | **4** | **1** | **12** | **12** | **0.71** | **MDR** |
|  | **19** | **9** | **1** | **7** | **7** | **0.41** | **MDR** |
|  | **22** | **6** | **1** | **10** | **10** | **0.59** | **MDR** |
|  | **30** | **7** | **3** | **7** | **7** | **0.41** | **MDR** |
|  | **37** | **10** | **1** | **6** | **6** | **0.35** | **MDR** |
|  | **38** | **11** | **1** | **5** | **5** | **0.29** | **NDR** |
|  | **39** | **4** | **2** | **11** | **11** | **0.65** | **MDR** |
|  | **40** | **13** | **0** | **4** | **4** | **0.24** | **NDR** |
|  | **45** | **6** | **0** | **11** | **11** | **0.65** | **MDR** |
|  | **46** | **4** | **2** | **11** | **11** | **0.65** | **MDR** |
|  | **67** | **12** | **2** | **3** | **3** | **0.18** | **NDR** |
|  | **81** | **8** | **0** | **9** | **9** | **0.53** | **MDR** |
|  | **91** | **8** | **0** | **9** | **9** | **0.53** | **MDR** |
|  | **101** | **10** | **1** | **6** | **6** | **0.35** | **MDR** |

**Supplementary Table 8. MAR index classification of the recovered isolates**

| **MAR index classification** | **Human** | | | **Feline** | | | **Total**  **(46)100%** |
| --- | --- | --- | --- | --- | --- | --- | --- |
|  | **Apparently Healthy**  **(2)%** | **Clinically Diseased**  **(23)%** | **Total**  **(25)100%** | **Apparently Healthy**  **(7)%** | **Clinically Diseased**  **(14)%** | **Total**  **(21)100%** |  |
| **NDR** | 1 (50%) | 12(52.2%) | 13(52%) | 3(42.9%) | 3(21.4%) | 6(28.6%) | 19(41.3%) |
| **MDR** | 1 (50%) | 10(43.5%) | 11(44%) | 3(42.9%) | 11(78.6%) | 14(66.7%) | 25(54.4%) |
| **XDR** | 0 (0%) | 0 (0%) | 0 (0%) | 1(14.3%) | 0 (0%) | 1(4.8%) | 1(2.2%) |
| **PDR** | 0 (0%) | 1(4.4%) | 1 (4%) | 0 (0%) | 0 (0%) | 0 (0%) | 1(2.2%) |
